# The complete mitochondrial genome of *Scyra compressipes* Stimpson, 1857 (Decapoda: Epialtidae)

**DOI:** 10.1080/23802359.2021.1899873

**Published:** 2021-03-18

**Authors:** Hyung-Eun An, Tae-June Choi, Chang-Bae Kim

**Affiliations:** Department of Biotechnology, Sangmyung University, Seoul, Republic of Korea

**Keywords:** *Scyra compressipes*, mitogenome, phylogeny, Epialtidae

## Abstract

The family Epialtidae is the most diversified family within superfamily Majoidea but there is no report for complete mitogenome of any species in this family. This study was performed to sequence a complete mitogenome of *Scyra compressipes* Stimpson, 1857, as the first mitochondrial genome report from the Epialtidae. The complete mitogenome sequence of *S. compressipes* was 16,415 bp and it consisted of 36 genes, including 13 protein-coding genes (PCGs), two rRNA genes, and 21 tRNA genes excluding tRNA-Leu (UAG). tRNA genes ranged from 63 bp to 72 bp in length. The base composition of a complete mitogenome of *S. compressipes* is 34.7% A, 15.3% C, 10.2% G, and 39.8% T. The phylogenetic position of *S. compressipes* in the superfamily Majoidea was examined based on 13 PCGs. The phylogenetic analysis showed that *S. compressipes* was most closely related to *Maguimithrax spinosissimus*, a representative of the family Mithracidae.

The family Epialtidae comprising approximately 100 genera and 500 species is the most diversified family within the superfamily Majoidea (Tavares et al. 2017). In spite of species diversity of the family, there is no report of mitochondrial genome from the representative species of the family until now. As a member of the Epialtidae, *Scyra compressipes* Stimpson, 1857, was recorded from Korea and Japan (Oh and Ko 2010). In this study, we provided a complete mitogenome sequence of *S. compressipes* and examined the phylogenetic position of the species in the molecular phylogeny of the superfamily Majoidea based on amino acid sequences of 13 protein-coding genes (PCGs).

An adult specimen of *S. compressipes* used in this study was collected from the offshore area of Namhae-gun, Gyeongsangnam-do, Korea (34°44′29.4″N, 128°02′54.0″E) in 9 October 2020. The specimen of *S. compressipes* was preserved in absolute ethanol and deposited in the Department of Biotechnology, Sangmyung University, with voucher number SMU00222. The genomic DNA of the specimen was prepared from gills using Qiagen DNeasy Blood & Tissue Kit (Qiagen, Hilden, Germany) according to the manufacturer’s protocol and sequenced on MGISEQ 2000 (MGI, Shenzhen, China). For assembly and annotation, MITObim ver. 1.9.1 (Hahn et al. 2013) and MITOS (Bernt et al. 2013) have been used, respectively. ARWEN ver. 1.2.3 (Laslett and Canbäck 2008) and tRNAscan-SE ver. 2.0.7 (Chan and Lowe 2019) were used for prediction of tRNA structure. The phylogenetic tree was reconstructed based on concatenated amino acid sequences of 13 PCGs with RAxML ver. 8.2.11 (Stamatakis 2014) by using the maximum-likelihood (ML) method with 1000 bootstrap replicates and MrBayes ver 3.2.7 (Huelsenbeck and Ronquist 2001) by using Bayesian inference (BI) analysis with 1,000,000 generations and sampling frequency of 1000 generations.

The complete mitochondrial genome of *S. compressipes* (GenBank accession number: MW451225) was 16,415 bp in length and contained 34.7% A, 15.3% C, 10.2% G, and 39.8% T nucleotide distribution. It consisted of 36 genes, including 13 PCGs, two rRNA genes, and 21 tRNA genes. Of 13 PCGs, nine of PCGs, including *cox1, cox2, cox3, cytb, nad2, nad3, nad6, atp6*, and *atp8* were encoded on H-strand while four PCGs, including*, nad1 nad4, nad4l*, and *nad5* were encoded L-strand. All PCGs had start codon as ATN (nine of ATG, two of ATA, one of ATC and ATT) and most of PCGs had stop codon as TAA except *cox1, cox3* (incomplete stop codon as T), and *nad2* (stop codon as TAG). rRNA genes were 1376 bp and 810 bp in length for 16S rRNA and 12S rRNA, respectively. Twenty-one tRNA genes ranged from 63 bp (tRNA-Ala) to 72 bp (tRNA-Val) in length. Although we searched secondary structures for the whole mitogenome sequences using ARWEN (Laslett and Canbäck 2008) and tRNAscan-SE (Chan and Lowe 2019), tRNA-Leu (UAG) was not found. For confirmation of the absence of the tRNA, new primer sets were designed for PCR and resequencing was executed by Sanger sequencing method. The experiments reconfirmed the absence of tRNA-Leu (UAG) gene in the mitogenome.

The phylogenetic analysis was performed based on the amino acid sequences of 13 PCGs in mitogenomes of six representative species from superfamily Majoidea: *S. compressipes* (GenBank accession number: MW451225), *Maguimithrax spinosissimus* (=*Damithrax spinosissimus*) (KM405516), *Chionoecetes japonicus* (MT750295), *Chionoecetes opilio* (MT335860), *Maja crispata* (KY650651), *Maja squinado* (KY650652), and two species from the superfamily Bythograeoidea: *Austinograea alayseae* (JQ035660) and *Gandalfus puia* (KR002727) and two species from the superfamily Portunoidea: *Scylla paramamosain* (FJ827761) and *Portunus trituberculatus* (AB093006) as outgroups. The molecular phylogeny suggested that *S. compressipes* clustered with *Maguimithrax spinosissimus*, a representative of the Mithracidae (Figure 1). The molecular phylogenetic relationship between families in the superfamily Majoidea presented in this study was consistent to previous studies (Hultgren and Stachowicz 2008; Jeong et al. 2020; Kim et al. 2020).

**Figure 1.**
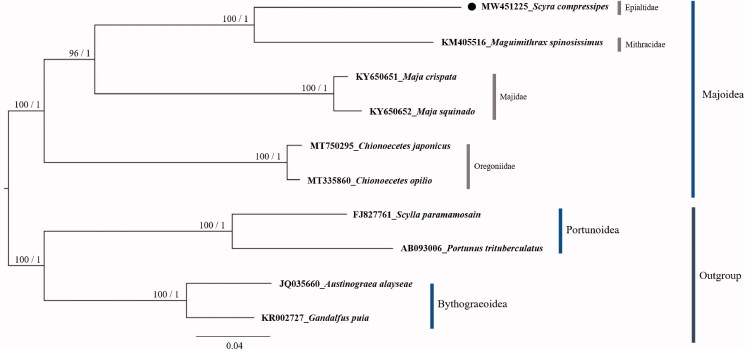
The phylogenetic tree of Majoidea. The bootstrap value (left) above 70% in the ML method and posterior probability (right) above 0.90 from the BI analysis were indicated at each node. GenBank accession number and scientific name for each species are shown at branch tips. The species reported in this study, *Scyra compressipes* is marked with a circle. The species names were used according to the World Register of Marine Species (http://www.marinespecies.org/).

## Data Availability

The genome sequence data that support the findings of this study is openly available in GenBank of NCBI at https://www.ncbi.nlm.nih.gov/ under the accession no. MW451225. The associated BioProject, SRA, and Bio-Sample numbers are PRJNA694515, SRR13555153, and SAMN17526022, respectively.
